# Subaxillary Replacement Flap Compared with the Round Block Displacement Technique in Oncoplastic Breast Conserving Surgery: Functional Outcomes of a Feasible One Stage Reconstruction

**DOI:** 10.3390/curroncol29120736

**Published:** 2022-11-30

**Authors:** Paolo Orsaria, Antonella Grasso, Georgeta Soponaru, Francesca Carnevale, Virginia Scorsone, Edy Ippolito, Francesco Pantano, Matteo Sammarra, Claudia Piccolo, Michele Altomare, Giuseppe Perrone, Vittorio Altomare

**Affiliations:** 1 Department of Breast Surgery, Campus Bio-Medico University, 00159 Rome, Italy; 2 Department of Radiation Oncology, Campus Bio-Medico University, 00159 Rome, Italy; 3 Department of Medical Oncology, Campus Bio-Medico University, 00159 Rome, Italy; 4 Department of Radiology, Campus Bio-Medico University, 00159 Rome, Italy; 5 Department of Trauma and Acute Care, Metropolitano Niguarda Hospital, 20162 Milan, Italy; 6 Department of Anatomical Pathology, Campus Bio-Medico University, 00139 Rome, Italy

**Keywords:** breast cancer, oncoplastic surgery, displacement, replacement, cosmetic outcomes

## Abstract

Background: For selected women diagnosed with breast cancer (BC), partial reconstructive techniques involve displacement or replacement procedures to improve cosmesis without compromising oncological safety. This study aims to evaluate the surgical outcomes of the round block (RB) compared with the subaxillary flap (SF) technique for patients with upper outer tumor. Patients and Methods: Thirty-three patients treated with oncoplastic conserving surgery (15 RB and 18 SF) were enrolled in this retrospective study. After carrying out a comparison of baseline characteristics, all cases were recruited for postoperative evaluation of oncological and cosmetic parameters. Moreover, we investigated several scoring combinations to check whether they could discriminate surgeon and patient satisfaction according to different functional results. Results: Median age (*p* < 0.05), average tumor size (*p* > 0.05), estimated resection volume (*p* > 0.05), and nodal involvement (*p* > 0.05) were slightly higher in the SF group. A greater frequency of DCIS (*p* < 0.05) in the RB series correlated with reintervention for positive margins (*p* > 0.001). At a mean follow-up of 19 months, no locoregional recurrences were recorded and early and late complications were comparable (*p* > 0.05). The overall satisfaction with cosmesis was characterized by similar proportions of good results (*p* > 0.05), with some details more related to each procedure. Conclusion: The proposed techniques represent effective solutions for reshaping that follows upper outer wide excision, achieving comparable complication rates, low reinterventions, and good aesthetic results in relation to technical and social functioning evaluations. However, it is crucial to establish a careful patient selection in order to manage correct surgical planning while predicting any potential sequelae or complication.

## 1. Introduction

Partial breast reconstructive techniques are increasingly being used in breast cancer (BC) surgery to improve cosmesis and patient satisfaction without compromising the oncological outcome.

These procedures involve reshaping or volume replacement to fill the parenchymal defect after tumor excision, potentially reducing the incidence of margin involvement, while respecting body image and breast parameters [1].

The advantage of performing a unilateral or bilateral approach is debated. Frequently, a very large resection may be taken using “therapeutic mammoplasty” and the chances of incomplete lumpectomy are very small. However, several reduction techniques may require a contralateral tissue redistribution to achieve symmetry, thus not limiting the number of surgical procedures [2].

In this regard, clarifying the indications of the modern oncoplastic conserving surgery might be based upon the predicted volume to be excised for any given cancer relative to breast size, while improving the preoperative assessment of the patient’s expectation [3].

Furthermore, an increasing demand for reduced scars has led to the development of numerous minimal or “invisible” incision procedures [4]. Among these options, the round block (RB) reconstruction is a useful method for resection of centrally located breast malignancies, especially for all patients with small defects and less than 20% of volume removal. It is a technically challenging operation associated with a wide skin sparing dissection to manipulate tissue through a periareolar doughnut incision. The mobilization of the gland is a key component of breast reshaping after tumor excision, especially in cases with dense tissue, moderate hypertrophy, and no severe ptosis [5]. The radial closing of the parenchymal defect to recone the breast, not only favours the ability to perform a re-excision of margins compared to the wise pattern procedure, but also a low rate of subsequent contralateral surgery with excellent symmetry at follow-up [6].

As opposed to the breast reshaping, the replacement repair is based on flaps outside the breast to restore the defect, as in case of subaxillary, thoracolateral, thoracoepigastric, bilobed, and myocutaneous flaps [7]. These procedures are most appropriate for patients with small-to-medium sized breasts, who cannot afford to lose the volume associated with displacement techniques or wish to avoid contralateral surgery.

In this regard, the subaxillary dermocutaneous fat flap (SF) is a novel approach for reconstruction of the upper outer quadrant and can be used if no more than 25% of tissue is removed for tumor clearance. After that, a wide local excision is carried out through the lateral mammary crease, the de-epitheliazed flap can be rotated and transposed to fill the defect [8]. Even if very thin patients with little subcutaneous fat are not suitable, using this procedure, the shape and symmetry of the conserved breast is well maintained and a contralateral procedure is seldom necessary [9].

There is limited evidence in the literature on the safety outcomes of different approaches in oncoplastic conserving surgery, but future horizons are likely to see the development of better selection tools to allow the best personalized strategies, while providing data on how patients feel about their management.

The purpose of this retrospective study is to review a single institution’s experience of round block repair compared with the subaxillary replacement flap for patients with upper outer quadrant BC, in order to assess surgical, oncological, and aesthetic outcomes related to these different partial reconstructions. 

## 2. Patients and Methods

A total of 33 patients with upper outer BC underwent oncoplastic conserving surgery using the RB or the SF technique at the Academic Breast Unit of Campus Bio-medico University Hospital of Rome, from January 2018 to December 2022. Patients were eligible if they were older than 18 years, had a histological diagnosis of invasive or in situ BC within 3 months, had a primary tumor deemed technically appropriate for surgical resection, and had no clinical evidence of distant metastatic disease. We did not exclude patients based on tumor size, age, lymph node status, or previous primary systemic treatment. All patients underwent a multidisciplinary approach, involving medical and surgical oncologists, plastic surgeons, breast radiologists, and radiation oncologists. In order to perform a breast conserving procedure, we localized the lesions the day before surgery using ultrasound examination; the sentinel lymph node (SN) was localized preoperatively by injecting Tc99m-nanocoll. Axillary lymph node dissection, chemotherapy, radiotherapy to the breast, and endocrine therapy were performed to our standard protocols if required. All patients had regular postoperative follow-up after any adjuvant treatment provided. The medical records including tumor characteristics, pathologic reports, complications, and surgeon or patient satisfaction assessed by a questionnaire were evaluated. All patients treated at our Institute provided informed written consent (ethical statement n. 07.19 PAR V ComET CBM).

The indications for displacement or replacement partial reconstruction surgery and study eligibility were based on excision volume, tumor location, glandular density, breast size, and level of ptosis.

## 3. Surgical Techinique

The resection was planned preoperatively, and markings were made with the patient in the upright position. The success of the procedure depended on patient selection and careful intraoperative management to perform an individual “customized” repair. 

**Figure 1:**
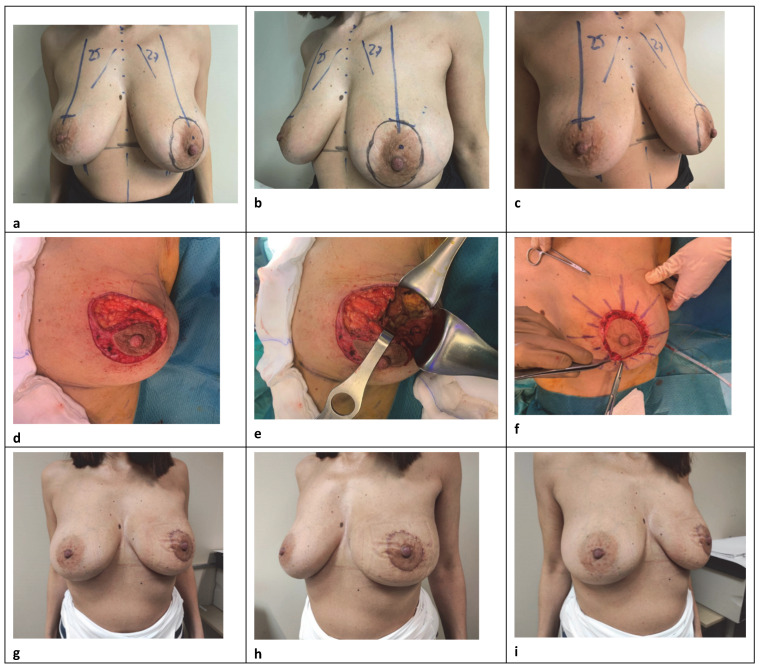
A 46-year-old woman with left multifocal invasive ductal carcinoma (pT1N1, luminal A G1). (**a**–**c**) Preoperative markings are shown. (**c**,**d**) Segmental resection of the upper outer on the left using the round block technique. (**e**,**f**) On table results after closing defects. (**g**–**i**) One month after surgery, frontal and side view.

The central round block approach is a volume displacement reconstruction with a varying amount of skin adjustement to consider depending on the volume loss or skin reduction requirement in patients with small-to-medium-sized breasts, with low to moderate ptosis, and who may not require contralateral surgery for symmetrization (Figure 1). It is a versatile technique that requires sophisticated glandular reshaping, while limiting scars with good aesthetic results. A circumareolar incision down to the dermis delimiting the outline of the areola, and another parallel concentric or oval not far away from the first, is made considering the location of the tumor and any potential asymmetry with the contralateral breast. A further trick to avoid scar widening and changes in areolar shape is to keep the width of the donut skin excision as close as possible, ideally within 20 mm [10]. After de-epithelializing the intervening ring area, slightly wider in the quadrant opposite to the resection to prevent deviation of the nipple–areolar complex (NAC), the dermis is cut up to 180° at the lesion side to provide a good exposure. The NAC remains vascularized by its posterior glandular base through the underlying plexus and the Würinger septum. The adjacent breast skin is widely undermined in the mastectomy plane between the subcutaneous fat and parenchyma at the level of the superficial fascia, preserving subdermal vascularization and trying to limit the dissection to the tumor-bearing quadrant. Up to a half breast dissection is enough to obtain a good operative field for wide excision and tissue reapproximation, thus decreasing the operation time or the possibility of postoperative seroma [11]. Excision of a radially oriented ellipse of parenchyma-facilitated closure follows, after eventually mobilizing the gland in the prepectoral plane. A surgical clip is inserted into the excised cavity, subsequently, to facilitate radiation therapy. No tight 2–0 absorbable sutures were used to close the defect radially and recone the breast tissue, often over a drain according to the extension of reshaping. In our approach, reducing to the maximum the periareolar skin removal, we used simple interrupted inverted dermal 3–0 sutures to support mild skin tension and simply maintain a good circular form. Running absorbable suture with 4–0 were used to secure the skin edge in an attempt to lower the chances of scarring. An SN biopsy was often performed through a separate transverse skin incision. This procedure was indicated for cases whose excision volumes were 10–20% of the total [12] and assumed to be best suited for treating periareolar lesions. Patients with very large and fatty breasts or a lot of additional skin with peripheral tumor location were not considered for this approach, nor for an extensive dual plane dissection. Our goal was to limit the breast detachment as much as possible to maximize the vitality of the glandular flaps and to ensure a better conical shape. Concerning the skin, the trend was to limit the amount of the periareolar de-epithelialization in order to prevent complications such as bad scarring and flattening owing to excess tension in the periareolar area, while achieving the best symmetry with the contralateral markings. However, careful attention to patient selection with good anatomic conditions, as well as to some operative details were critical in performing this procedure.

**Figure 2:**
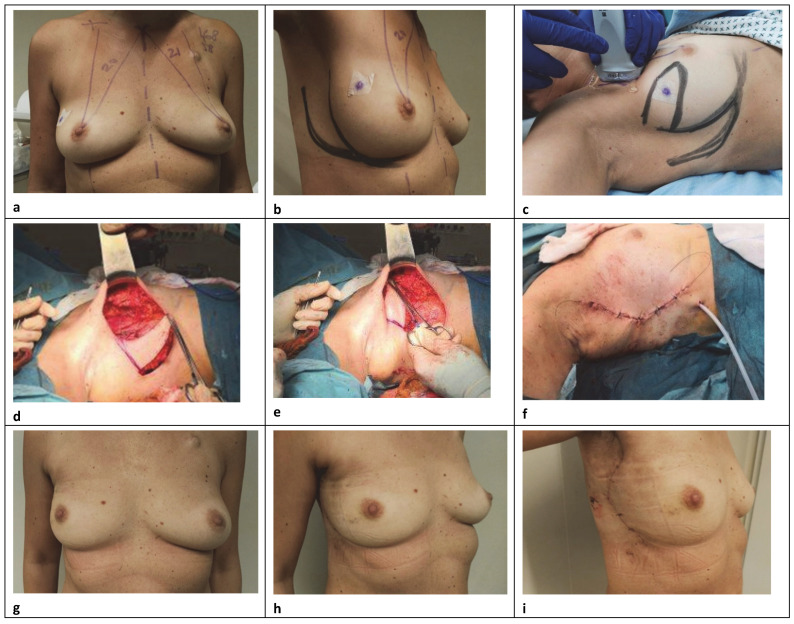
A 45-year-old woman with right invasive ductal carcinoma (yT0N0, Triple Negative G3). (**a**–**c**) Preoperative markings are shown. (**c**–**e**) Segment resection of the upper outer part on the right using the subaxillary replacement flap technique after neoadiuvant chemotherapy. (**e**,**f**) On table results after closing defect. (**g**–**i**) One month after surgery, frontal and side view.

For the replacement repair, the technique basically employs a transposition flap of skin and subaxillary fat for the breast defect, using the excess of subcutaneous autologous tissue in the lateral extramammary thoracic region (Figure 2) [9]. Its major clinical application is in patients who refuse higher morbidity procedures or are not candidates for more extensive reconstruction, particularly with myocutaneous flaps. The main indication for this local flap was primarily in patients with small-volume breasts with or without ptosis and included cases with moderate lateral defect where there was not enough breast tissue to perform the reconstruction by local glandular flaps or reduction mammoplasty techniques [13]. For this purpose, a convex flap design was used that, rather than depend on an axial blood vessel for nourishment, relied upon the dermal-subdermal plexus of blood vessels. A lateral contour acces is performed and the parenchyma of the breast upper part is widely mobilized under the skin and above the muscle to the tumor level, in order to perform the lumpectomy. A full-thickness glandular resection is performed, and the adequacy of surgical margins is evaluated by intraoperative macroscopic assessment. Usually, a rhomboid or a wedge-shaped flap was designed on the subaxillary region and the amount of tissue available was determined by a pinch test. The base of the flap varied from 4 to 9 cm and the length from 6 to 10 cm, respectively. For small defects, the flap is planned as a triangle located exclusively on the axilla. For moderate and large excisions, the distal limit can reach the whole lateral thoracic region, designing the inferior and superior limits more obliquely and with curved borders. A surgical clip is inserted into the excised cavity, subsequently, to facilitate radiation therapy. The skin and subcutaneous axilla fat are dissected from the underlying muscles in a medial direction. Then, this “tissue bridge” is rotated into the lateral breast quadrant and fixed with no tight 2–0 absorbable sutures to the chest wall or breast parenchyma to close the resection cavity. The defect is close under the skin after being de-epitheliazed or with skin replacement when necessary, and the contour is restored by subcutaneous axillary adipofascial tissue with preservation of perforating vessels, in order to ensure a sufficient blood supply coming to the lateral part of the partial reconstruction [4]. The wound is closed in two layers with 3–0 and 4–0 absorbable sutures, respectively, over a drain, producing a neat single curvilinear scar in the lateral mammary crease, with mild skin tension and good circular form according to this anatomical subunit. An SN biopsy is performed through the same skin incision in all cases. All patients with superolateral tumors are potential candidates for subaxillary flap reconstruction, although some limitations to this procedure exist in thin patients, with insufficient tissue to replace the volume removed and reduce the deformity to an acceptable level. In this regard, it is important to allow optimal positioning of the axillary incisions to avoid scar widening or an ischemic flap with subsequent necrosis [14]. Moreover, an important requirement for applying a correct approach should consider that the primary tension of the closure never have to displace a neighboring structure and the prominent landmarks, especially the nipple and the inframammary fold. 

## 4. Study Design and Conduct

All cases were reprospectively recruited for postoperative evaluation of oncological and cosmetic outcomes after any oncoplastic treatment, including also early and late surgical complications. 

The size of the tumours was measured and classified according to the American Joint Committee on Cancer (8th edition) staging criteria, i.e., T1a–b (<10 mm), T1c (11–20 mm), T2 (21–50 mm), or T3 (>50 mm) [15]. Nuclear grade and axillary node metastases were also examined by histopathology. The intrinsic BC subtypes were identified according to the clinicopathological criteria recommended by the 2013 St. Gallen International Expert Consensus Report [16]. The patients were categorized based on the receptor status of their primary as follows: luminal A [oestrogen receptor-positive (OR+) or progesterone receptor-positive (PR+) and HER2–]; luminal B HER2– (OR+, HER2–, and at least one of Ki-67 “high” or PR “negative or low”); luminal B HER2+ (OR+, HER2 overexpressed or amplified, any Ki-67 value, any PR); HER2 (OR– or PR– and HER2+); or triple-negative (OR– or PR– and HER2–). Tumours were considered HER2-positive only if they were scored as 3+ by immunohistochemistry (IHC; strong, complete membrane-staining in >10% of cancer cells) or showed HER2 amplification (ratio >2) using fluorescence in situ hybridization (FISH). In the absence of positive FISH data, tumours scored as 2+ on IHC were considered negative for HER2. Tumours were also classified as luminal or nonluminal according to hormone receptor expression. The primary disease was classified as multifocal at the time of initial diagnostic work-up if the radiologist or histological assessment available after surgery described two or more lesions separated by ≥1cm of normal parenchyma. Furthermore, in our institution, we routinely measured the estimated breast volume resected, using a method previously described by Behluli et al. In brief, after each primary surgical intervention, we approximated the resection volume from the dimensions of the specimens using the following formula: V = a × b × c, where V was the resection volume (cm^3^), and a, b, and c were the specimen length (cm) in the medial/lateral, superior/inferior, and anterior/posterior directions, respectively [17]. The tumor volume was estimated to be a cube, based on the formula: V = L3, where V was the resection volume (cm^3^), and L was the maximum histological lesion diameter.

The oncological endpoint was evaluated by rate of positive or close margins, defined by the presence of cancer cells less than 2 mm from the specimen’s margins at the histological examinations. The tumor and specimen volumes were compared with the rate of margin involvement and need of further surgery, either re-excision or mastectomy. Complete data on the oncological outcomes of local recurrence, metastasis, and overall survival were also reported. 

Postoperative complications were grouped as major (wound dehiscence, adipose necrosis, areola–nipple complex necrosis), minor (infection, seroma, hematoma), and late (fat necrosis, fibrosis, hypertrophic scar) [18].

At the time of the cosmetic evaluation, all patients had completed their postoperative radiation therapy. The surgeon’s technical opinion and patient self-assessment were achieved by rating the shape and symmetry between the treated and untreated breast, also, with respect to the position on the nipple–areola complex, the perception of the surgical scar, and appraisal of their cosmetic outcome and satisfaction. All parameters were evaluated on a 3-point scale (excellent, good, and fair) based on these five items [8]. The observer evaluated patients’ photographs, taken at their last follow-up appointment, in frontal views with arms in the neutral position on the hips, with arms raised, and in profile. Quality of life outcomes after surgery were further described using a questionnaire based on the following criteria assessed by the patients: psychological wellness with social functioning, physical discomfort, and side effects of adjuvant radiotherapy [19]. Finally, we compared group A (round block: RB) and group B (subaxilllary replacement flap: SF) for all the selected outcome variables, in order to assess the operative measures and quality outcome variables for each type of surgery performed.

## 5. Statistical Analysis

Data originated from patient dossiers, including electronic access and review of operation and pathology reports, as well as discharge letters. Continuous variables were summarized using mean, median, and standard deviation, categorical variables in percentile. Furthermore, the associations between the type of surgery and clinicopathological factors (age, invasive tumor size, nuclear grade, lymphovascular invasion, ER, PR, and HER2 status, molecular subtypes, and Ki-67 labeling index) were examined. For hypothesis testing, we applied the two-tailed t-test and chi-squared test followed by Fisher’s exact test for confirmation. Statistical difference was considered significant for *p* < 0.05. All statistical and stratified analyses were performed using IBM SPSS 23 software (IBM, SPSS Statistics, Chicago, IL, USA).

## 6. Results

The series included 33 patients who underwent oncoplastic breast conserving surgery for unilateral upper outer BC, of which 15 were treated with the round block technique and 18 with the subaxillary replacement flap. 

Table 1 shows the demographic information of the patients and their primary tumor characteristics.

The median age at the surgery was 58 years (range = 34–80), 53 in the RB (range = 34–73) and 62 (range = 44–80) in the SF, respectively (*p* = 0.022). Of all the cases, 69.6% (*n* = 23/30) were peri- or postmenopausal (*p* = 0.701). 

The average tumour size was 1.7 cm (range = 0.6–4.5 cm), with no significant difference between groups (RB: 1.5 ± 0.7 cm vs. SF: 1.8 ± 0.9 cm; *p* = 0.712). The primary disease was classified as multifocal in 33% (*n* = 11/33), of which it was almost half of the cases in the RB (46%, *n* = 7/15) compared to the SF group (22%, *n* = 4/18; *p* > 0.001), and 6% (*n* = 2/33) received neoadjuvant chemotherapy (all in the SF; *p* = 0.168).

The most common histotype in the primary tumor was invasive ductal carcinoma (23/33 = 70%), followed by invasive lobular carcinoma (6/33 = 18%), and ductal carcinoma in situ (DCIS) (4/33 = 12%). A DCIS diagnosis was significantly higher in RB cases (4/15 = 26.6%; *p* = 0.02).

Furthermore, a ductal in situ component was associated with invasive disease in less than half of the invasive cases (13/29 = 44.8%), while for the defining nuclear grades, 18.2% (*n* = 6) were scored as G1, 48.5% (*n* = 16) as G2, and 33.3% (*n* = 11) as G3.

The incidence of luminal and nonluminal infiltrating tumours was 83.3% and 16.7%, respectively. Most patients had luminal A (14/30 = 46.7%), followed by luminal B HER2− (9/30 = 30%), HER2-positive luminal B (2/30 = 6.7%), nonluminal HER2-positive (1/30 = 3.3%), and triple-negative (4/30 = 13.3%). 

Overexpression of c-erbB-2 was found in 3/31 cases (9.7%) and 13 patients (13/31 = 41.9%) had a high Ki-67 expression (≥20%), significantly higher in the SF compared to the RB group (10/18 = 55.5% vs. 3/12 = 25%; *p* < 0.001). 

Most DCIS were over 10 mm with prevailing hormone receptor positivity (3/4 = 75%).

Twenty-six (78.8%) of the thirty-three were staged as pN0, and seven (21.2%) had ipsilateral axillary LN metastases, with a significant major proportion of axillary dissection in the subaxillary (6/18 = 33.3%) compared to the round block series (1/15 = 6.7%; *p* = 0.001). N stage was categorized by the staging system of the American Joint Committee on Cancer (8th edition): 6 patients (18.2%) were in N stage 1, 1 (3%) in N stage 2, and none in N stage 3 (15). There were 5 patients (15.2%) in stage 0, 17 (51.5%) in stage I, 8 (24.2%) in stage II, and 3 (9.1%) in stage III. In this context, the sentinel node biopsy (SNB) was performed in only 87.9% (29 of 33), while the axillary dissection rate was 21.2% (7 of 33), 1 in the RB (14.3%) and 6 among SF cases, respectively (85.7%).

Postoperative radiotherapy was administered in all patients. Adjuvant chemotherapy was recommended for 8 patients (24.2%), 3 in RB (20%) and 5 in the SF cases (27%; *p* = 0.043). All patients with hormone-receptor (HR) positive tumors (28/33 = 84.8%) were subject to receive endocrine therapy, 13 (86.6%) in RB and 15 in SF (83.3%; *p* = 0.625).

The median follow-up duration was 19 months (range 4–36).

## 7. Oncologic Outcome

The mean resection volume was slightly larger in the subaxillary replacement cases than for the round block (SF: 128.9 ± 89.4 cm^3^ vs. RB: 107.2 ± 75.8 cm^3^), although the distribution did not statistically differ (*p* = 0.158).

Regarding the approximated tumor volume, we observed no significant differences between groups (SF: 7.6 ± 8.8 cm^3^ vs. RB: 7.2 ± 8.1 cm^3^) even in consideration of the dimensional homogeneity of the cases included (*p* = 0.612).

We reported a higher re-excision rate in the RB (2/15 = 13.3%) than in the SF series (0/18 = 0%), where no further resection was revealed to be necessary (*p* > 0.001). All positive margin patients had a previous DCIS diagnosis. Only one patient underwent contralateral reduction in the RB group after a second re-excision due to extensive disease.

No locoregional recurrences were observed at the end of the follow-up, and only three percent (1 of 33) developed distant relapse and subsequent BC related death (Table 2). This patient had G3 triple negative invasive ductal carcinoma and showed disease progression with brain recurrence after primary systemic treatment and complete pathological response (ypT0N0) subsequent to subaxillary replacement repair and axillary dissection.

## 8. Postoperative Complications

Overall, there was a 3% rate of major complications, all in the subaxillary flap group (2/18 = 5.5%), exclusively represented by delayed healing of the surgical wound (*p* = 0.374).

An incidence of 18.2% (*n* = 6/33) of minor complications was reported, 11.1% (*n* = 2/18) in the SF and 26.6% (*n* = 4/15) in the RB group (*p* < 0.001), characterized mainly by the onset of seroma or hematoma in the postoperative period, with a higher trend in the displacement (19.9%) compared to the replacement technique (5.5%).

Onset of late complications (hypertrophic scar, adipose necrosis, fibrosis) was recorded in 24.2% (*n* = 8/33), 27.7% (*n* = 5/18) in the SF and 20% (*n* = 3/15) in the RB cases (*p* = 0.697). However, in the first group, late complications were represented exclusively by the appearance of hypertrophic scar (27.7%), while in the second group by fibrosis and fat necrosis (16.6%), (Table 3).

All major and minor complications were managed conservatively and responded to outpatient treatment with local wound care. No unanticipated readmission or return to the operating room were documented. No cases of surgical site infection were reported, considering that a short-term antibiotic prophylactic therapy (5 days) was prescribed to all patients. 

## 9. Cosmetic Outcome and Patient Satisfaction

Aesthetic results were evaluated following radiotherapy in 29/33 patients after excluding 4 cases (two in each group) for incomplete data (Table 4 and Table 5). The overall satisfaction with cosmetic outcome assessed by the patient was considered excellent in 8/16 (50%) in the SF, while it was 6/13 (46%) in the RB series (*p* = 0.220); the overall satisfaction assessed by the surgeon was considered excellent in 8/16 (50%) in the SF, while in 10/13 (77%) in the RB series (*p* = 0.09).

There were no significant differences in shape, breast, and NAC symmetry between patients and surgeon assessment with similar proportions of good and poor outcome in both the series (*p* = 0.541). A higher significant trend was reported while measuring the scar appearance where the overall satisfaction rate evaluated by the surgeon encountered excellent results in 8/13 (62%) in the RB compared to 2/16 (13%) in the SF group (*p* = 0.001).

Among the quality of life outcomes assessed by the patients after surgery (Table 6), psychological wellness and social functioning was evaluated with a maximum score in 75.8% (*n* = 22/29) of the cases included in the study: 81.3% (*n* = 13/16) in the “Subaxillary rotation flap” and 69.2% (*n* = 9/13) of the “Round block” group, respectively (*p* > 0.05).

Furthermore, a persistent postoperative pain, skin tension in the treated breast, ipsilateral arm impairments or signs of lymphedema were considered as representative elements of physical discomfort. In this context, a minimum score was reported by 72.4% (*n* = 21/29), 81.3% (*n* = 13/16) in the SF and 61.5% (*n* = 8/13) in the RB (*p* = 0.41).

Moreover, the appearance of thickening, redness, skin dehydration, or pain were recorded to evaluate the adjuvant radiotherapy outcomes with the related potential side effects.

In this case, a minimum score was reported by 71.4% (*n* = 21/29), 75% (*n* = 12/16) of patients in the SF and 69.3% (*n* = 9/13) in the RB group (*p* = 0.440).

## 10. Discussion

Correct surgical planning means offering a variable number of projects with heterogeneous complexity, but at the same time predicting and managing any sequelae or complications that may result from that type of treatment. In this context, it is crucial to establish a careful selection of patients who can be potentially submitted to conservative management, based on disease location, glandular or fatty volume composition, and breast size, always doing measurements regarding symmetry and ptosis degree [20]. Furthermore, the aim to maintain a good cosmetic result is directly linked to a wide excision with radical margins and being able to prevent local recurrences or disease progression [21].

The present study aims to compare two different reliable approaches for upper outer quadrantectomies both respecting oncological safety and preserving a pleasant breast contour. The analysis focuses not only on the patient’s clinical or pathological features, but also on the percentage of complications and satisfaction after surgery, to implement the selection criteria for these techniques. Even if both groups revealed homogenous characteristics (Table 1), there was a slight significant trend for the round block repair in younger patients, confirming that this procedure is applied more in breasts with higher glandular density and lower ptosis [13]. 

On the contrary, the subaxillary rotation flap has been more frequently indicated in cases with an average age of 62 up to 80 years, also in consideration of a greater frequency of adipose morphology or associated comorbidities [22]. 

Although a significant difference in resection volumes was not reported, we noticed a tendency towards greater excisions in the replacement approach, which can improve a better oncological radicality in outermost tumors, considering even the chance of removing the overlying skin if necessary. However, the patient’s morphology is always determinant in establishing the amount of tissue available in the lateral thoracic region to fill the cavity defect, and often, this treatment is not applicable in young and thin patients [23]. 

A greater resection efficiency of the replacement technique must also be correlated to a larger tumor size in this group and with a significantly higher nodal involvement. In all these cases, the axillary dissection was possible from the same incisional pattern used to set up the flap which, despite the greater scar compared to the round block, was able to offer an excellent locoregional control on the neurovascular bundles [24]. Moreover, in line with a more aggressive biological profile, in the SF group, there was a significantly higher cut-off of ki67 when categorized as ≤20%, and for >20%, an increased frequency of luminal B HER2+ or TN BC and a larger application of primary systemic treatment.

In general, these techniques often allow for wider local tumor excision, potentially reducing the incidence of margin involvement, while enhancing the cosmetic outcome [25].

However, in accordance with the hypothesis of a lower volume of resection in the RB group, as an intrinsic limit of the procedure in our hands, all cases of re-excision for positive margins occurred in these patients, and always for a DCIS diagnosis [14]. In this context, several authors assessed that subaxillary dermocutaneous fat flaps can be used in all patients, regardless of breast size, if no more than 25 per cent of the breast tissue is removed for tumour clearance [26]. On the other hand, the proportion of resection volume achieved with the round block approach is usually slightly inferior (up to 20% of tissue loss), always considering a technical procedure that potentially should not require a contralateral approach. Thus, moderate-to-large-sized breasts tend to exhibit poor outcomes due to asymmetrical breast size caused by a shrinking volume if the excision volume is >20%, plus potential problems of late-onset scar widening or changes in the areola shape, and in this case, the round block approach should be considered in combination with other techniques [27]. 

Moreover, from this analysis, it is highlighted that the two pathways could have a different spectrum of complications. For instance, the longer incisional pattern of the subaxillary flap was more subject, not only to a higher occurrence of delayed healing in the early postoperative period but also to a more frequently hypertrophic long-term scarring [28]. These features could represent the basis for optimizing the surgical technique, accurately defining the tension lines during the partial breast reconstruction and the wound management in the follow-up.

On the other hand, the onset of seroma or hematoma was the most frequent complication of the round block, probably linked to the extensive dual-plane undermining achieved by subcutaneous and prepectoral dissection. These minor events could be related in the long term with development of fat necrosis and fibrosis, thus potentially decreasing the overall aesthetic outcome. To better manage the risk of these problems, the background breast composition must be carefully evaluated by preoperative mammograms, since most of fat necrosis occurs with a higher frequency in the low-density tissue group with a major fatty composition [29]. Moreover, in these higher risk patients, the surgical technique may possibly include a “cold dissection” (with scissors or blade), to decrease the tissue damage caused by the electrocautery and if alternative oncoplastic approaches are not feasible.

Finally, we did not observe any significant difference in terms of cosmetic outcomes between the two groups, which can act as evidence that both the procedures represent a safe and effective solution for the reshaping that follows upper outer resection with comparable patient and surgeon satisfaction [30,31].

The major scarring consequences in the subaxillary cases had a negative impact on the overall assessment both from a subjective or technical point of view. For this reason, more recent interest in “short” scar procedures has prompted breast surgeons to reduce complications more carefully, while always providing for more aesthetic and long-lasting shapes [32]. However, this concept must not compromise the technical choice to obtain an adequate shape based on the type of patient, although this can result in more complex incisional patterns. Furthermore, the fact that psychosocial well-being was judged to be slightly better in the replacement group may be motivated by the assumption that the scar is not directly visible in the frontal position when the patient looks in the mirror, resulting in a lower cognitive impact. Therefore, the patient’s evaluation is important in determining the quality of cosmetic outcome after oncoplastic surgery [30]. Indeed, the cultural and emotional aspects elaborated by every single patient, also based on their own social development, might explain why, in this study, the patient assessment was often characterized by lower scores than those obtained from the specialist’s technical evaluation [31].

In conclusion, to decrease breast deformities, it is strongly suggested to think in terms of aesthetic subunits during partial resections and reconstructions. Meticulous handling of tissue is essential to avoid necrosis, decreased sensation, lymphedema, and unnecessary undermining, which might violate perforators. Moreover, the breast size, shape, and degree of ptosis must be considered when discussing the potential for poor cosmetic results and designing the best reconstructive option, since unreasonable expectations are best managed before surgery, as dealing with them postoperatively can be difficult [32]. 

Consequently, we must properly evaluate the case to select the recommended surgical procedure and to be able to obtain reproducible results, since to be an oncoplastic surgeon is a great responsibility, the patient’s life and hopes being in our hands. 

This study presents the limitation of being retrospective and a longer follow-up could further confirm the oncological and cosmetic safety of the proposed surgical approaches. Furthermore, the ratio of the patient’s original breast volume to the excised tissue cannot be evaluated since the breast volume was not measured. In this regard, the resection volume in comparison with the tumor size could also have been inaccurate, because their assessment was conventionally performed through a mathematical model and not through clinical or instrumental evaluation.

Another drawback concerns the relatively small number of cases and, moreover, this analysis did not adress the complexities of selection, which clearly involves many factors including both patient and physician concerns and biases. As such, our protocol should be considered exploratory in nature. Optimally, we would have had sufficient cases to evaluate the impact of different clinicopathological criteria in determining the surgical treatment, but further studies with increased numbers and a longer follow-up may ascertain potential differences between these parameters.

In summary, our study has shown that the proposed procedures represent safe and effective solutions for reshaping following upper outer breast wide excision, achieving comparable complication rates, low reinterventions for positive margins, and good aesthetic results in relation to technical and social functioning evaluations.

In this context, further analyses may allow more patients to be considered for this multiparametric comparison and could provide relevant meaningful information and longer prognostic data.

## Figures and Tables

**Table 1:** Patient demographics and baseline characteristics.

	Subaxillary Rotation Flap (*n* = 18)	Round Block (*n* = 15)	Total (*n* = 33)	*p* Value
Age, median (range), years	62 (44–80)	53 (34–73)	58.5	*p* = 0.022
*<50 anni*	4 (22%)	6 (40%)	10 (30%)	*p* = 0.701
*>50 anni*	14 (78%)	9 (60%)	23 (70%)	
Clinical tumor size, median (range), cm	1.8 (0.6–4.5)	1.5 (0.7-3)	1.7 (0.6–4.5)	*p* = 0.712
*<20 mm*	11 (61%)	11 (73%)	22 (67%)	
*≥20 mm*	7 (39%)	4 (27%)	11 (33%)	
Multifocal	4 (22%)	7 (46%)	11 (33%)	*p* > 0.001
Histologic type				
*IDC*	15 (83%)	8 (53%)	23 (70%)	*p* = 0.452
*ILC*	3 (17%)	3 (20%)	6 (18%)	*p* > 0.05
*DCIS*	0	4 (27%)	4 (12%)	*p* < 0.02
DCIS component in invasive carcinoma	8 (44%)	5 (33%)	13 (39%)	*p* = 0.012
Grade				
*I*	3 (17%)	3 (20%)	6 (18%)	*p* = 0.058
*II*	10 (55%)	6 (40%)	16 (49%)	*p* = 0.029
*III*	5 (28%)	6 (40%)	11 (33%)	*p* = 0.24
Receptor Status				
*Luminal A*	7 (39%)	7 (58%)	14 (47%)	*p* = 0.516
*Luminal B HER 2+*	7 (39%)	2 (17%)	9 (30%)	*p* = 0.003
*Luminal B HER 2−*	1 (5%)	1 (8%)	2 (7%)	*p* = 0.595
*Nonluminal HER 2+*	0	1 (8%)	1 (3%)	*p* = 0.448
*Triple negative*	3 (17%)	1 (8%)	4 (13%)	*p* = 0.116
Ki67 > 20%	10 (56%)	3 (25%)	13 (43%)	*p* < 0.001
Lymph node status				
*pN0*	12 (67%)	14 (93%)	26 (79%)	*p* = 0.413
*pN1*	5 (28%)	1 (7%)	6 (18%)	*p* = 0.676
*pN2*	1 (5%)	0 (0%)	1 (3%)	*p* = 0.419
*pN3*				
Axillary surgery				
*SLND*	14 (77%)	15 (100%)	33 (100%)	nc
*ALND*	6 (33%)	1 (8%)	7 (21%)	*p* < 0.001
Neoadjuvant therapy	2 (11%)	0	2 (6%)	*p* = 0.168
Adjuvant Therapy				
*RT*	18 (100%)	15 (100%)	33 (100%)	nc
*CT*	5 (27%)	3 (20%)	8 (24%)	*p* = 0.043
*HT*	15 (83%)	13 (87%)	28 (85%)	*p* = 0.625

**Table 2:** Oncological and surgical outcomes.

	Subaxillary Rotation Flap (*n* = 18)	Round-Block (*n* = 15)	*p* Value
Specimen Volume, median (range) ± DS, cm^3^	128.9 ± 89.4	107.2 ± 75.8	*p* = 0.158
Histological tumor Volume, median (range) ± DS, cm^3^	7.6 ± 8.8	7.2 ± 8.1	*p* = 0.612
Re-excision rate	0	2 (13%)	*p* > 0.001
LRR	0	0	nc
Metastasis	1 (5%)	0	nc

**Table 3:** Postoperative complications.

	Subaxillary Rotation Flap (*n* = 18)	Round Block (*n* = 15)	Total (*n* = 33)	*p* Value
MAJOR COMPLICATIONS	1 (5%)	0	1 (3%)	*p* = 0.374
Delayed healing or dehiscence	1 (100%)	0	1 (3%)	
Nipple–areolar necrosis	0	0		
Fat necrosis	0	0		
MINOR COMPLICATIONS	2 (11%)	4 (27%)	6 (18%)	*p* < 0.001
Infection	0	0		
Hematoma	1 (50%)	2 (50%)	3 (9%)	
Seroma	1 (50%)	4 (100%)	5 (15%)	
LATE COMPLICATIONS	5 (28%)	3 (20%)	8 (24%)	*p* = 0.697
Fat necrosis	0	2 (67%)	2 (6%)	
Breast fibrosis	0	3 (100%)	3 (9%)	
Hypertrophic scar	5 (100%)	0	5 (15%)	
Pseudoptosis	0	0		

**Table 4:** Aesthetic results—surgeon’s assessment.

	Subaxillary Rotation Flap (*n* = 16)				Round Block (*n* = 13)			
	Excellent	Good	Fair	*p* Value	excellent	Good	Fair	*p* Value
SHAPE	10 (63%)	6 (37%)	0		11 (85%)	0	2 (15%)	*p* > 0.05
NAC SYMMETRY	9 (56%)	7 (44%)	0		9 (69%)	4 (31%)	0	*p* > 0.05
BREAST SYMMETRY	7 (44%)	6 (37%)	3 (19%)		8 (62%)	4 (31%)	1 (7%)	*p* > 0.05
SCAR	2 (13%)	12 (75%)	2 (12%)		8 (62%)	5 (38%)	0	*p* < 0.001
OVERALL SATISFACTION	8 (50%)	8 (50%)	0		10 (77%)	1 (8%)	2 (15%)	*p* = 0.09

**Table 5:** Aesthetic results—patient’s assessment.

	Subaxillary Rotation Flap (*n* = 16)				Round Block (*n* = 13)			
	Excellent	Good	Fair	*p* Value	Excellent	Good	Fair	*p* Value
SHAPE	8 (50%)	8 (50%)	0		5 (38%)	6 (46%)	2 (16%)	*p* > 0.05
NAC SYMMETRY	6 (38%)	9 (56%)	1 (6%)		7 (54%)	5 (38%)	1 (8%)	*p* > 0.05
BREAST SYMMETRY	8 (50%)	5 (31%)	3 (19%)		6 (46%)	6 (46%)	1 (8%)	*p* > 0.05
SCAR	5 (31%)	7 (44%)	4 (25%)		5 (38%)	6 (46%)	2 (16%)	*p* > 0.05
OVERALL SATISFACTION	8 (50%)	8 (50%)	0		6 (46%)	5 (38%)	2 (16%)	*p* = 0.220

**Table 6:** Quality of Life.

	Subaxillary Rotation Flap (*n* = 16)				Round Block (*n* = 13)			
	Max	Mid	Min	*p* Value	Max	Mid	Min	*p* Value
Psychosocial well-being	13 (81%)	3 (19%)	0		9 (69%)	4 (31%)	0	*p* > 0.05
Physical discomfort	0	3 (19%)	13 (81%)		0	5 (38%)	8 (61%)	*p* = 0.410
Radiotherapy side effect	0	4 (25%)	12 (75%)		1 (8%)	3 (23%)	9 (69%)	*p* = 0.440

## Data Availability

Data supporting results can be found in the Breast Cancer Surgery Department Dataset.
